# London Ambulances

**Published:** 1904-02-13

**Authors:** 


### Editor's Letter-Box.

[Our Correspondents are reminded that prolixity is a great bar to publication, and that brevity of style and conciseness of statement greatly facilitate early insertion.]

### LONDON AMBULANCES.

Sir Henry Burdett writes to the *Times* on the 8th inst.:—I gather from the leading article in the *Times* of the 1st inst. that a misapprehension prevails as to the present provision of ambulance accommodation for the removal of street accidents in London. In your leading article you mention the Hospitals Ambulance Association by name, but have evidently no particulars of its work. This street ambulance system was established in 1891 through Mr. H. L. Bischoffsheim, who provided the large initial cost for supplying the ambulances, and who has ever since maintained the system and paid all the outgoings attached to it. These ambulances have been employed for the removal of street accidents since the establishment of the service in 1889 no less than 22,314 times. At the present time the "Bischoffsheim Ambulance Service" has 56 stations, at each of which there is at least one ambulance, *i.e.*, fire-brigade stations 10, hospitals 15, cab ranks and central thoroughfare stations 25, and other stations, like the Royal Exchange, 6. When I last looked into the figures for the purpose of giving evidence before the London County Council I found that from one-half to two-thirds of the street accident cases in London were removed by the Bischoffsheim ambulances.

The point I wish to make is that this system was established at the cost of Mr. Bischoffsheim some 16 years ago, when I represented to him the importance of such a provision for London. All efforts to secure the establishment of a service through municipal or other authorities have failed. These efforts mostly originated with this service, like the movement you refer to, which happened ten years ago, and also a subsequent inquiry by a special committee of the London County Council. Mr. Bischoffsheim has behaved with the most generous liberality throughout, and I have his authority to state that he is prepared, as he has always been, to supply every requisite, even horse or motor ambulances, and indeed to do anything in reason to secure to London a properly equipped and perfectly organised ambulance service.

At the same time Mr. Bischoffsheim is equally ready to hand over this work to the London County Council, or to any public authority, provided that authority will formulate a practical scheme and guarantee that it shall be maintained in efficiency for the future.

.·. Dr. Arthur James published a letter in the "British Medical Journal," of the 6th inst., under the heading "The Metropolitan Street Ambulance Association." That letter contained no intimation of the existence of the Street Ambulance branch of the Hospitals Association, nor of the St. John's Ambulance work. The Hon. Secretary of the Street Ambulance Association, Mr. Thomas Ryan, is a neighbour of Dr. Arthur James's, and the facts in regard to this matter at the time he wrote his letter ought to have been within his knowledge. It is a growing fault of these latter days to fail to do justice to the services of those who, like Mr. Bischoffsheim, are content to do good public work quietly and without publicity.—Ed. The Hospital.

